# Combining roller crimpers and flaming for the termination of cover crops in herbicide-free no-till cropping systems

**DOI:** 10.1371/journal.pone.0211573

**Published:** 2019-02-07

**Authors:** Christian Frasconi, Luisa Martelloni, Daniele Antichi, Michele Raffaelli, Marco Fontanelli, Andrea Peruzzi, Paolo Benincasa, Giacomo Tosti

**Affiliations:** 1 Department of Agriculture, Food and Environment, University of Pisa, Pisa, Italy; 2 Department of Agricultural, Food and Environmental Sciences (DSA3), University of Perugia, Perugia, Italy; University of Helsinki, FINLAND

## Abstract

The termination of cover crops in conventional no-till systems is mostly conducted mechanically in combination with herbicides. Combining flaming and roller crimpers could be a viable solution to avoid using herbicides for cover crop termination in farming systems where herbicides are banned, or at least to reduce their use in an integrated management approach. This research tested the effects of flaming used in combination with three different types of roller crimpers to terminate a fall-sown cover crop mixture of winter pea and barley. The cover crop termination rate was visually assessed in terms of percentage of green cover provided by cover crop plants at different intervals from the termination date, and estimated using a log-logistic non-linear regression model with four parameters. Machine performance data are also reported. The results show that, irrespective of the roller type, flaming significantly boosted the effect of the roller crimpers. In fact, an economic threshold for cover crop suppression of 85% was reached only when the rollers were used in combination with flaming. Nevertheless, none of the methods were able to reach the 100% of cover crop suppression. In some case, the combined use of flaming and roller crimpers allowed reaching the 90% of cover crop devitalisation, which happened six weeks after the termination date. More importantly, the use of flaming in combination with rollers shortened the time needed to achieve the estimated levels of devitalisation, compared with the rollers used alone. We conclude that flaming is an effective tool to increase the effectiveness of roller crimpers. Nevertheless, further research is needed to identify solutions to overcome the barrier of the high operational costs of flaming, which is constraining its wider adoption by farmers. Future studies could focus, for instance, on the development of a new prototype of combined machine for crimping and flaming the cover crops simultaneously, which could potentially reduce the operational costs.

## Introduction

Cover crops are used in conservation systems to protect the soil from erosion, enhance water infiltration and storage, reduce nutrient losses through surface run-off or leaching, supply biologically fixed nitrogen, improve soil physical structure, increase organic matter content and compete with weeds, reducing the reliance on herbicides [1, 2, 3, 4, 5]. High biomass production is essential to ensure the maximum provision of such benefits from cover crops [6]. There are many cases in the literature where the cover crop is terminated prior to planting the cash crop [7]. The cover crop residues remain on the soil surface and act as a mulch that suppresses the weeds, also protecting the soil from rapid desiccation and keeping the soil moisture at good levels for cash crop seed germination or plant establishment [8].

In systems banning the use of herbicides (e.g., organic farming), an easy method to terminate cover crops in no-till systems is to use roller crimpers. Such rollers are not power take off propelled and thus can be operated at a relatively high speed [9]. A roller consists of a cylindrical drum with a variable number of blades of different shapes (e.g., wave-shaped, curved, straight, etc.) on the outer surface. When the roller is operating, the blades crimp the stems of the plants, thus terminating the cover crop [10]. The main action of roller crimpers is to crush, but not completely cut, the cover crop stems at equal intervals in the same direction that the following cash crop will be planted. Crimping the cover crop causes plant injury and accelerates senescence [1]. Ashford and Reeves [10] indicated that, when rolling was conducted at the appropriate plant growth stage, rollers were as effective as herbicides in terminating a number of cover crop species.

The use of roller crimpers has been tested and reported in an increasing number of papers [1, 8, 11, 12]. Most report a higher termination rate when roller crimpers are used at least from the flowering stage of legume cover crops and the anthesis of grass cover crops [13]. A delayed termination date of winter grown cover crops may result in postponed sowing dates of the spring cash crop. The delay in sowing date may cause significant yield loss in the spring cash crop, unless the water in the soil would not be a limiting factor, as it was the case of the study reported in Teasdale et al. [14]. Thus, a wider adoption of roller crimpers would likely pass through identifying solutions to improve their effectiveness even at earlier phenological stages of cover crops.

To improve the effectiveness of roller crimpers, several design issues first need to be considered [12]. The shape of the blades, for instance, could be adjusted in order to increase their sharpness or the number of contact points between the roller and a single stem of the cover crop. This latter solution may be preferable as it preserves the crimping effect whilst increasing the number of crimping points and accelerating sap loss [11]. One possibility to achieve this is through the replacement of continuous, parallel blades with fragmented and staggered types.

An alternative aimed at improving the termination efficiency of roller crimpers is to use herbicides after the rolling operation. In systems that allow the use of herbicides, these could be applied to speed up the termination of cover crop but also of weed plants, so that the following cash crop can be established at the optimal time, especially in case of adverse weather conditions (i.e. cold and wet spring) [11]. In organic or integrated low-input systems where the use of herbicides is banned or reduced, respectively, flaming could be a valid alternative to improve the effect of rollers and/or speed up the cover crop termination. Flaming makes it possible devitalizing cover crop plants without tilling the soil through the use of direct heat in the form of fire. The high temperature of the flame denaturises the plant proteins, without burning plants tissues, and thus desiccates them [15]. The desiccated cover crop biomass remains on the soil acting as mulch.

Bavougian et al. [8] tested flaming to terminate cover crops, but not in combination with roller crimpers. They tested roller crimpers and flaming as two different no-till methods to terminate cover crops. They found a high variability in the percentage of cover crop suppression (from 18% to 100%) with both methods depending on the cover species and time of termination.

To the best of our knowledge there has been no research combining the effect of rolling with flaming in order to devitalize cover crops and create mulches in no-till conservation systems. This research tests the effect of flaming used in combination with three different types of roller crimpers in order to terminate a winter grown cover crop mixture.

## Materials and methods

### Experimental set-up, design and treatments

The experiment was conducted in 2015 and 2016 at the experimental farm of the University of Perugia (FieldLab, Papiano, Perugia, Italy) (42°57'22"N 12°22'32"E, 165 m above sea level) on two neighbouring fields.

The soil was clay-loam (*Fluventic Haplustept*) in the top 0.5 m, with 46% silt, 34% clay, 20% sand, 1.2% organic matter (TOC = 9.3 g kg^-1^ and total N = 0.82 g kg^-1^), with a high content of extractable P (29.9 mg kg^-1^, Olsen method) and exchangeable K (258 mg kg^-1^), and pH_H2O_ = 7.8.

**Fig 1:**
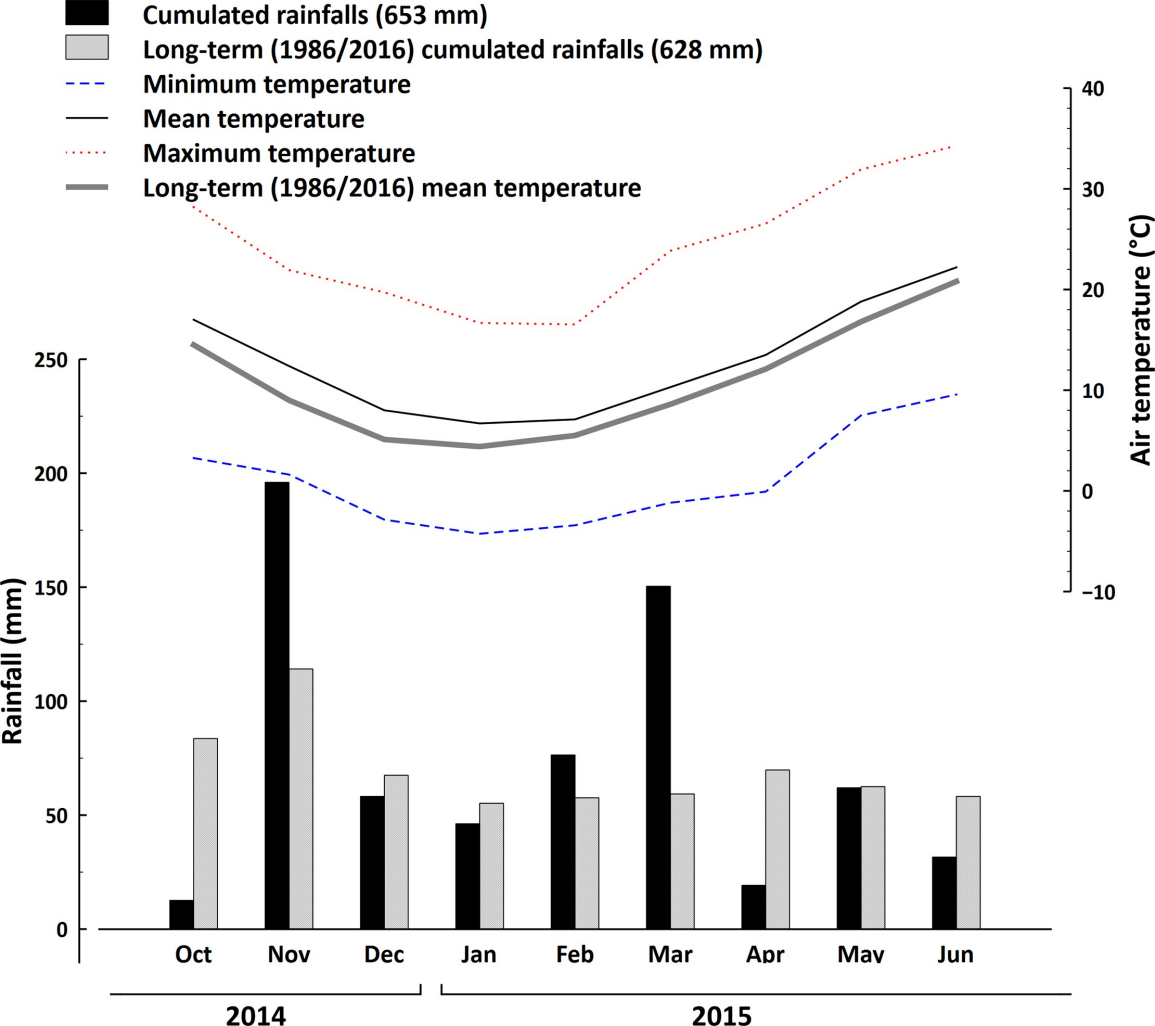
Monthly (October 2014 –June 2015) and 30-year cumulated precipitation, and monthly average, maximum and minimum and 30-year average temperature recorded by the meteorological station at FieldLab in Papiano (Perugia, Italy) (42°57'22"N 12°22'32"E).

**Fig 2:**
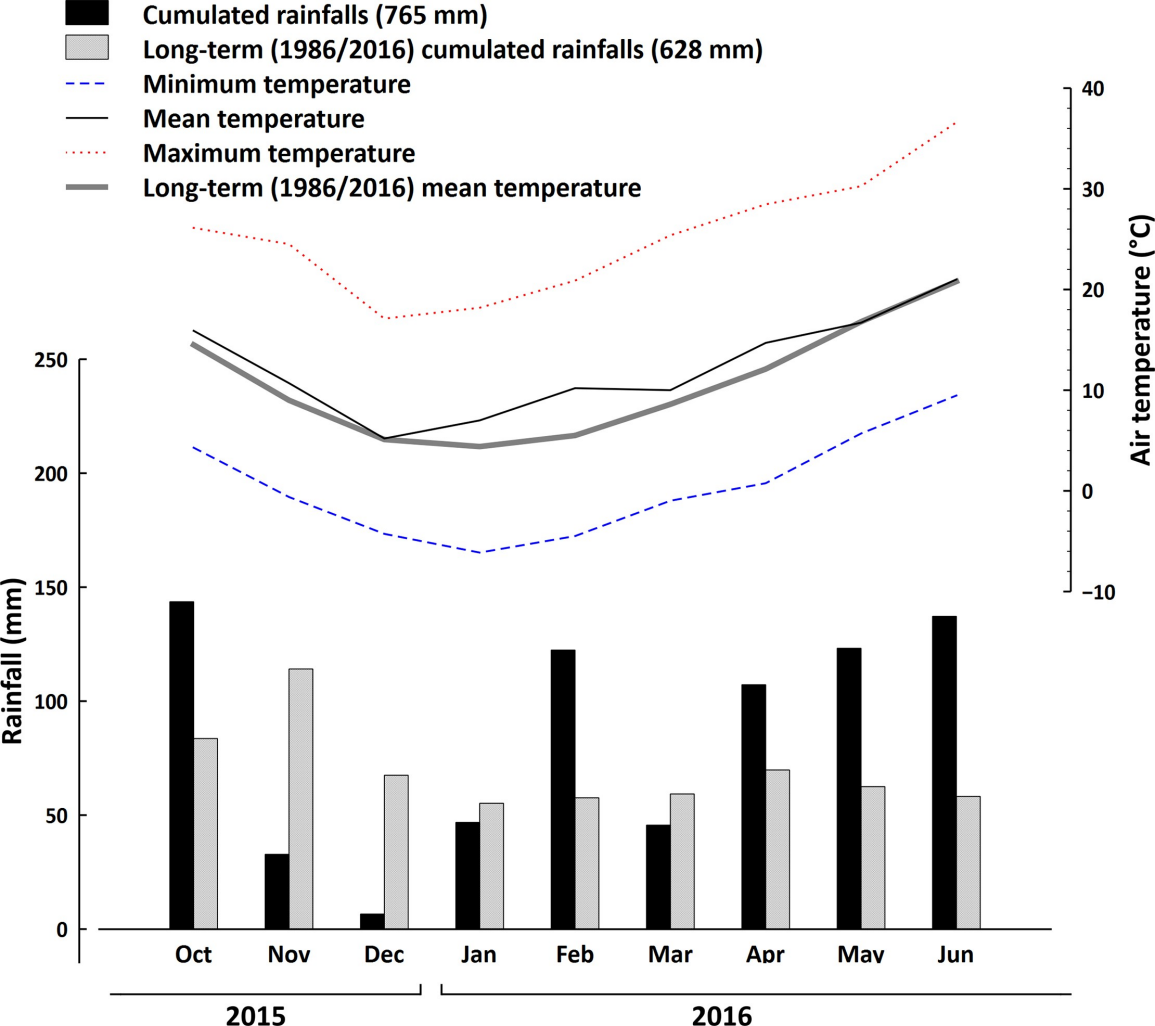
Monthly (October 2015 –June 2016) and 30-year cumulated precipitation, and monthly average, maximum and minimum and 30-year average temperature recorded by the meteorological station at FieldLab in Papiano (Perugia, Italy) (42°57'22"N 12°22'32"E).

The cover crop was a mixture of barley (*Hordeum vulgare* L. cv. “Asso”) and field pea (*Pisum sativum* ssp. *arvense* L. cv. “Arkta”). This mixture was chosen on the basis of previous studies carried out at the University of Perugia, which showed a high ecological complementarity between these two species in terms of both light and N resources, and a consequent high and constant biomass production of the mixture [16, 17]. The cover was sown in the two years on 31 October 2014 and on 26 October 2015, respectively. According to a replacement design [18], the adopted sowing rate of the mixture was 90 kg ha^-1^ for pea and 50 kg ha^-1^ for barley (i.e. 75% and 25% of the ordinary full sowing rates of the pure crops, respectively). In order to maximize species interaction and ensure a uniform plant distribution, barley was broadcast sown (S800, Finotto Srl, Venezia, Italy), and the pea was sown in rows using an air seeder (Vicon LZ301, Kverneland Group Italia, Mantova, Italy). In each year, the previous crop was common wheat (*Triticum aestivum* L.). After wheat harvest, the soil was ploughed (Dupao-30, SOGEMA Srl, Terni, Italy) at a depth of 25 cm, then disk-harrowed (28 FCI 230/FR, Nardi Spa, Perugia, Italy) at 15 cm depth and finally harrowed with a rotary harrow (DC3000, Maschio Gaspardo Spa, Padova, Italy) at 10 cm depth. In both years, the soil had been fertilized before tillage with 75 kg ha^-1^ of P_2_O_5_ as G27 (Panfertil Spa, Ravenna, Italy) and 75 kg ha^-1^ of K_2_O as potassium sulphate (Pastorelli Spa, Pavia, Italy). This was intended as an anticipated P-K fertilization of the cash crop, since these nutrients should better be incorporated into the soil while this would have been hampered in case of cover crop termination by roller crimping and in view of a no-till seeding of the following cash crop. The cover crop was not irrigated, and, thanks to the highly competitive effect of the mixture, weed control was unnecessary because no weeds were present until the termination dates. Figs 1 and 2 report the monthly minimum and maximum temperatures and precipitations at the experimental site in the two crop years, together with the 30-year mean values.

The growth stage of the cover crop at the time of termination was 59 BBCH for pea [19] and 61 BBCH for barley [20]. The cover crop termination was conducted on 22 April in 2015 and 19 April in 2016, using three different types of roller crimpers (i.e., Fleco, Clemens and Rodale). The same rollers were also used in combination with flaming, applied immediately after the rolling. All rolling and flaming operations were performed along the cover crops rows, thus the cover crop was flattened in one direction only. All the machines were coupled with a New Holland TL100 tractor. The Rodale roller was front mounted, the Clemens roller was back mounted, and the Fleco roller was towed.

A randomized split-plot design with four blocks was used. The three rollers (main plots) were used with or without flaming (subplots) for a total of 24 experimental units. The main plots were 20 m long and 3 m wide; sub-plots were 10 m long and 3 m wide.

### Characteristics of the rollers and the flaming machine

**Fig 3:**
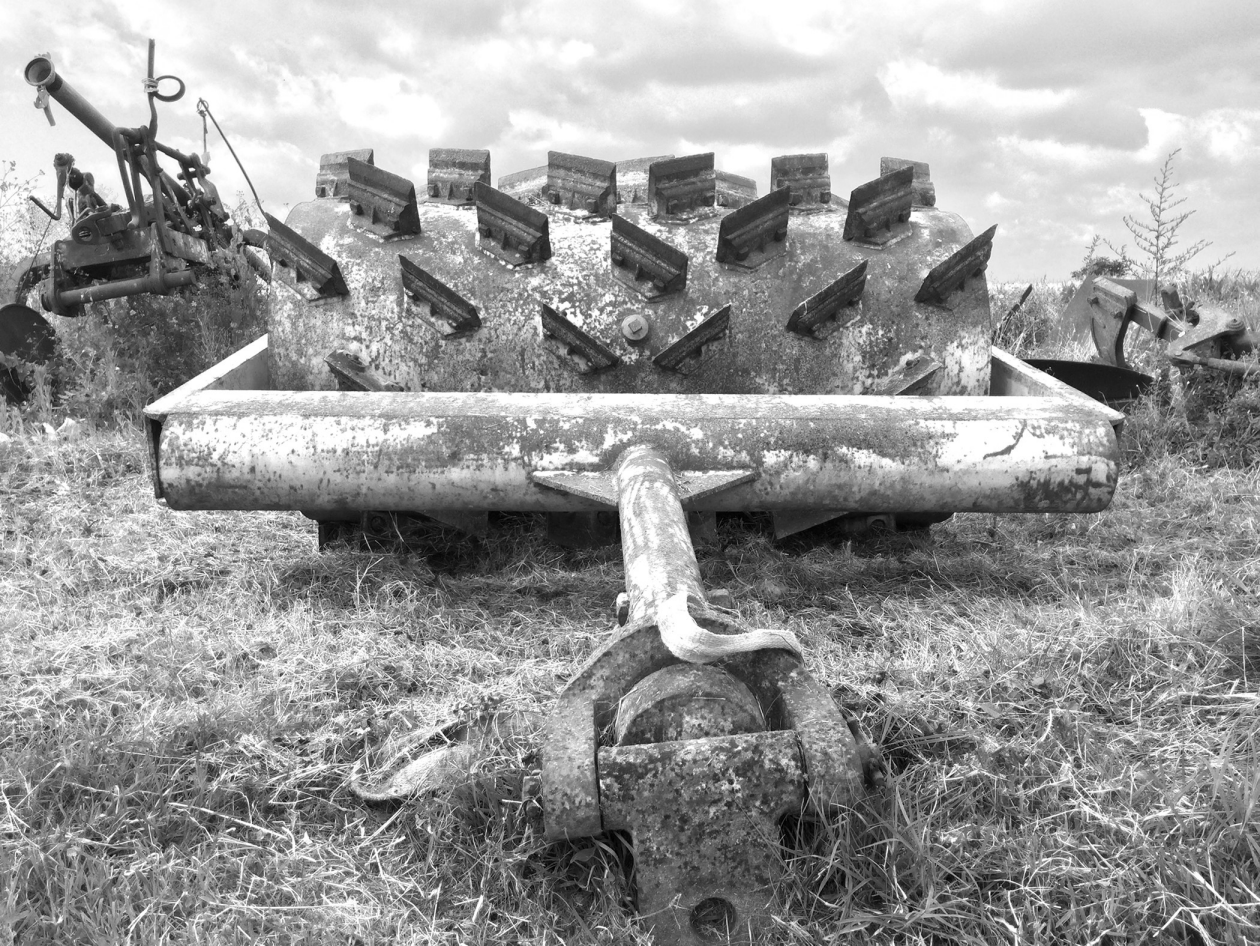
The Fleco heavy roller.

**Fig 4:**
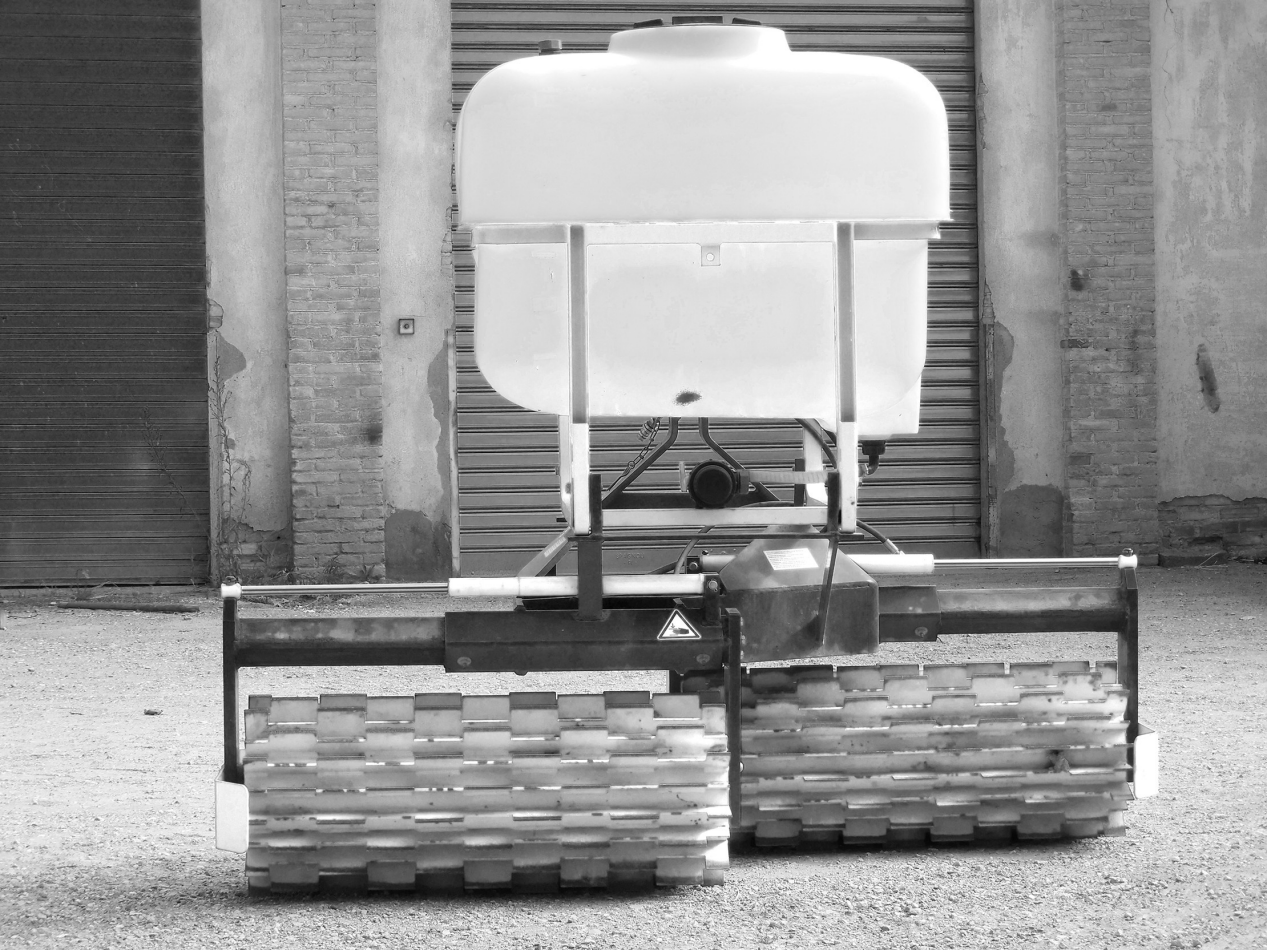
The Clemens Eco-roll with the Hexagon frame and water container.

**Fig 5:**
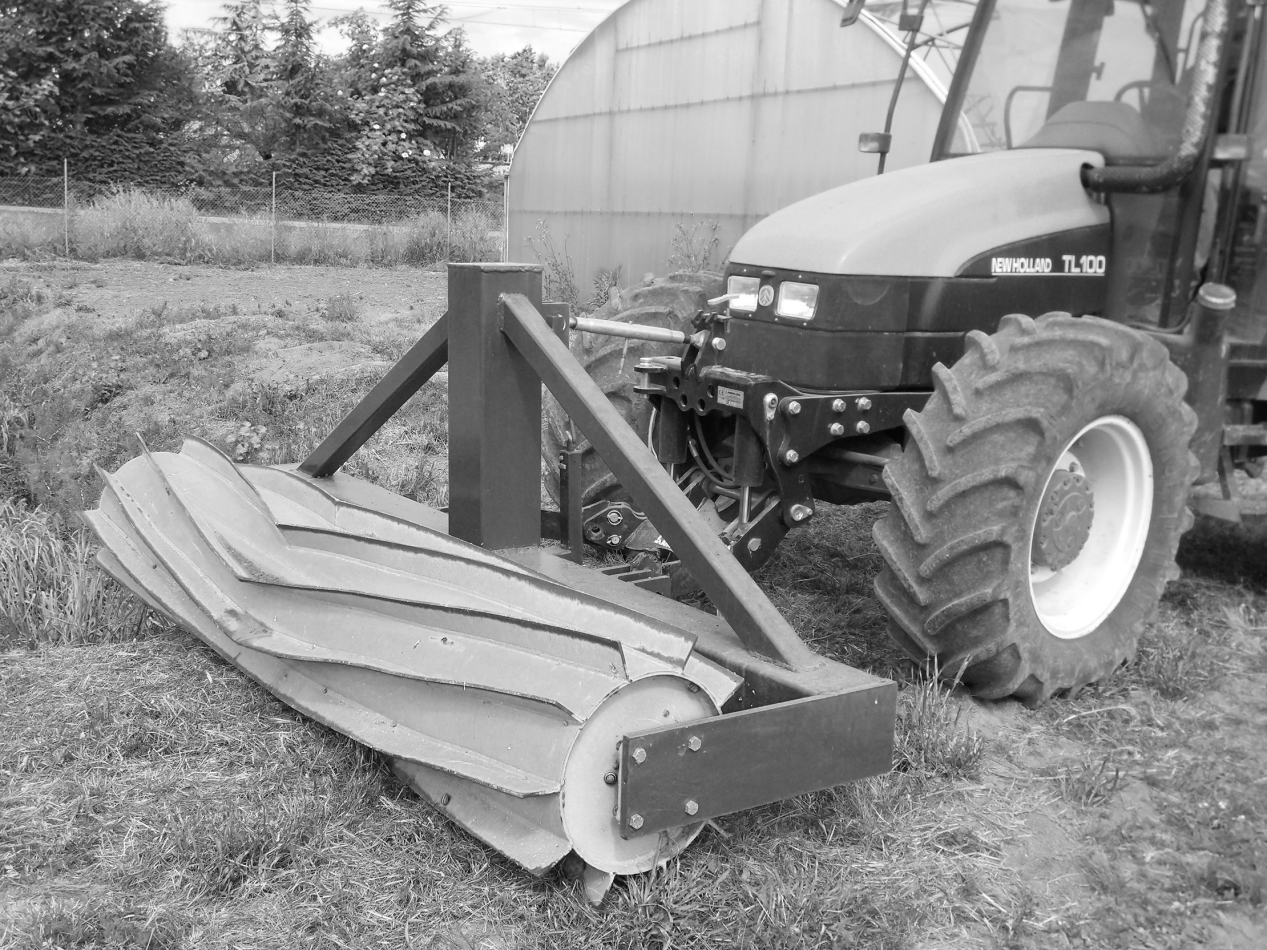
The Rodale Institute design-based roller crimper front-mounted at the New Holland TL100 tractor.

The main characteristics of the roller crimpers are reported in Table 1. The Fleco roller was a very old heavy roller constructed by the Fleco Corporation (Jacksonville, Florida, USA) in the 1940s (Fig 3). The Clemens roller was an Eco-roll type with the Hexagon frame (Clemens Technologies, Germany). It consisted of two cylindrical drums joined together. The minimum working width was 1.12 m and the maximum was 1.98 m. A tank containing a maximum of 300 L was mounted onto the frame (Fig 4). The Rodale roller crimper was custom built following the plans provided by the Rodale Institute [21] (Fig 5). The Clemens and Rodale rollers were used at maximum weight (i.e. completely filled), whilst and the Fleco roller was used empty.

**Table 1:** Roller crimpers main characteristics.

	Fleco	Clemens	Rodale
Cylinder units (No.)	1	2	1
Cylinder diameter (m)	0.95	0.28	0.42
Cylinder width (m)	1.82	1.00	2.88
Type of blades	Staggered-curved	Staggered-straight	Double-curved
Blade rows (No.)	12	20	10
Space between blade rows (m)	0.29	0.07	0.11
Blade length (m)	0.23	0.10	2.94
Blade height (m)	0.150	0.075	0.080
Blade thickness (m)	0.015	0.007	0.007
Weight of the empty roller (kg)	1700	650	650
Weight of the full roller (kg)	2900	950	950

**Fig 6:**
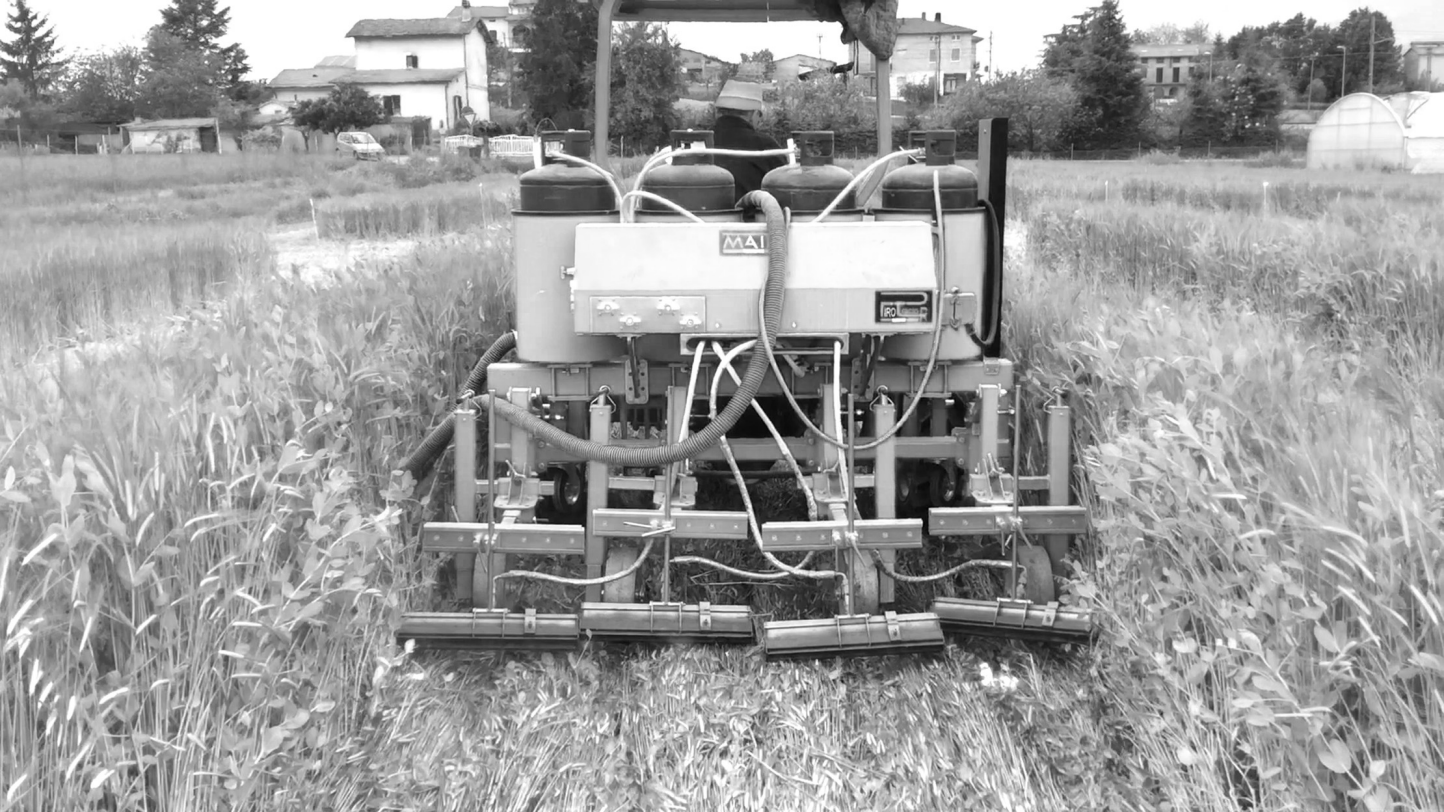
The flaming machine conducting cover crop mixture termination after the roller crimper operation.

The flaming machine was constructed by MAITO (MAITO Srl, Arezzo, Italy) based on a prototype designed and developed at the University of Pisa [22]. The flaming machine was equipped with four 50 cm wide rod burners for a total working length of 2 m (Fig 6). The liquefied petroleum gas (LPG) dose applied in this experiment was 68.27 kg ha^-1^, obtained by combining a forward speed of 1.15 km h^-1^ with a working pressure of 0.3 MPa. This LPG dose was chosen based on previous experience in the selection of LPG doses for weed devitalisation [23, 24, 25, 26]. The temperature of the flame was 1300 (±100)°C.

### Data collection

#### Machinery performance and costs

All the machine performance parameters were calculated. The ground pressure was calculated taking into account the ground support area when the roll was in a static position. The field efficiency (i.e. the ratio of the theoretical field time and the total time spent in the field) was computed by referring to a hypothetical area of 10,000 m^2^ (30.00 m wide and 333.33 m long). The theoretical field time is the time the machine is effectively operating at an optimum forward speed and performing over its full width of action. The total time for conducting the operation was calculated by summing the machine adjustment time (including plugging and unplugging), the theoretical field time, the turning time, and the time to refuel the tractor and/or replace empty LPG tanks. However, the travelling time back and forth the field was not included. The total cost per use was calculated by summing the fixed and variable costs for each of the three rollers and the flaming machine coupled with a New Holland TL100, following a standard methodology for cost determination [27]. The rate of depreciation was determined considering a purchase price of €46,445 for the New Holland TL100, €12,000 for the Fleco roller, €5,500 for the Rodale roller, €6,844 for the Clemens roller, and €11,407 for the flaming machine. The purchase cost of the Fleco roller (no longer available on the market) was chosen based on the cost of a similar roller used by Miyata et al. [28]. The purchase cost of the Fleco roller used by Miyata et al. [28] was reduced because of the higher weight (6391 kg) compared to that used in this experiment. Additionally, the purchase cost was adjusted based on the inflation rate from 1983 to 2015, then the resulting cost was converted from dollars into euros based on the 2015 annual average exchange rate.

The economic lifetime considered was 15 years for the rollers, 12 years for the tractor, and 10 years for the flaming machine. The repairing and maintenance factor was 80% for the tractor, 75% for the flaming machine, 70% for the Clemens roller, and 60% for the Rodale and Fleco rollers. The labour costs for the operator was 18 € h^-1^, and the LPG cost was 2.27 € kg^-1^.

#### Cover crop height and biomass

Cover crop biomass and height were collected the day before the termination date in both years. The height was measured randomly throughout each plot using a custom-made scale rod. Canopy height was measured considering the end of the spike (excluding beards) for barley and the last fully-expanded leaves for pea (5 measurements for each species in each plot were taken). The biomass was randomly sampled from the central part of each plot using a 1 m^2^ area frame. Species were kept separate, then sub-samples were oven dried for 48 h at 80°C, and the final dry biomass was reported as tonnes per hectare.

#### Cover crop termination

Due to the high biomass production of the cover crop mixture, the percentage of green cover before the termination was 100% in all plots in both years. The data on percentage green cover after termination were estimated from digital images using the IMAGING Crop Response Analyser [29] online software. The digital images were taken from an area of 0.25 m^2^ (50 cm × 50 cm) at 1, 7, 14, 21 and 42 days after the cover termination, always with the same geographical coordinates at the centre of the sub-plot, at the same distance between the cover crop and the camera, and avoiding shades by the use of an umbrella. The brightness of the digital images was equalized before analysis. The digital image analysis is described in Rasmussen et al. [30]. At the time of the termination there were no weeds in the plots. Few little weeds grown after termination, when present, were not separated from cover crop species. The percentage green cover consisted of all green aboveground vegetation because, in our opinion, the little presence of weeds did not bias the green cover assessment.

### Statistical analysis

Data normality was assessed using the Shapiro–Wilk test. Other tests consisted of the Student’s t-test to verify that the mean error was not significantly different to zero, the Breusch–Pagan test for homoscedasticity, and the Durbin–Watson test for autocorrelation.

Cover crop biomass and height data were modelled in a linear mixed model using the extension package lmerTest (Tests in Linear Mixed Effects Models) [31] of R software [32]. The year was the fixed factor and replicates the random factor. The analysis of variance was run.

A preliminary analysis regarding the percentage of green cover was conducted using a linear mixed model in the R extension package lmerTest [31]. Data were logit transformed. A combined variable/factors was constructed from the time (days after termination), type of roller (Fleco, Clemens and Rodale) and the flaming factor (whether flaming was applied or not after rolling), and then it was considered as a fixed factor. The other fixed factor was the year (2015 and 2016). The random factor was the replicates. The analysis of variance was run and the combined variable/factors, the year, and the interaction between the combined variable/factors and the year resulted significant. Data were thus analysed separately for each year using a model describing the relationship between the percentage of green cover and time (days after termination).

The percentage of green cover as affected by time (days after termination) and by the interaction between the type of roller (Fleco, Clemens and Rodale) and the flaming factor (applied or not after rolling) was modelled using a four-parameter log-logistic nonlinear regression (Eq 1):

$$Y=c+\frac{\left(d-c\right)}{\left\{1+exp[b(logX-loge)]\right\}}$$ (1)

where (*Y*) is the response (percentage green cover), (*c*) is the percentage green cover at the lower limit of the curve, (*d*) is the percentage green cover at the upper limit of the curve, (*b*) is the slope of the curve at the inflection point, (*X*) is the predictor (time), and (*e*) represents the time with 50% of green cover between the upper and the lower limit of the curve (i.e. at the inflection point). The lack-of-fit test indicated that the four-parameter log-logistic model was comparable to a one-way ANOVA model using an approximate F test, meaning that the nonlinear regression provided an acceptable description of the data. A significance test to compare the four-parameter log-logistic model and the simple linear regression model with slope 0 (a horizontal regression line corresponding to no time effect) was conducted. The resulting p-value = 0.00 indicated that there was a highly significant effect of the time and of the interaction between the type of roller and flaming factor on the green soil cover. Absolute effective times with 50% and 15% of green cover were estimated from the curves. The drc (Dose-Response Curves) R extension [33] was used to fit the nonlinear regression model, to estimate the parameters, and to plot the regression curves.

The comparisons between pairs of estimated values were computed by estimating the 95% confidence interval of the difference between the values (Eq 2):

$$CI(difference)=(x_{1}-x_{2})\pm 1.96\sqrt{{\left({SE}_{X_{1}}\right)}^{2}+{\left({SE}_{X_{2}}\right)}^{2}}$$ (2)

where (*x*_1_) is the mean of the first value, (*x*_2_) is the mean of the second value, (*SEx*_1_) is the standard error of (*x*_1_), and (*SEx*_2_) is the standard error of (*x*_2_) [34].

If the resulting 95% confidence interval (CI) of the difference between values did not cross the value 0, the null hypothesis that the compared values were not different was rejected.

## Results and discussion

### Cover crop termination

**Fig 7:**
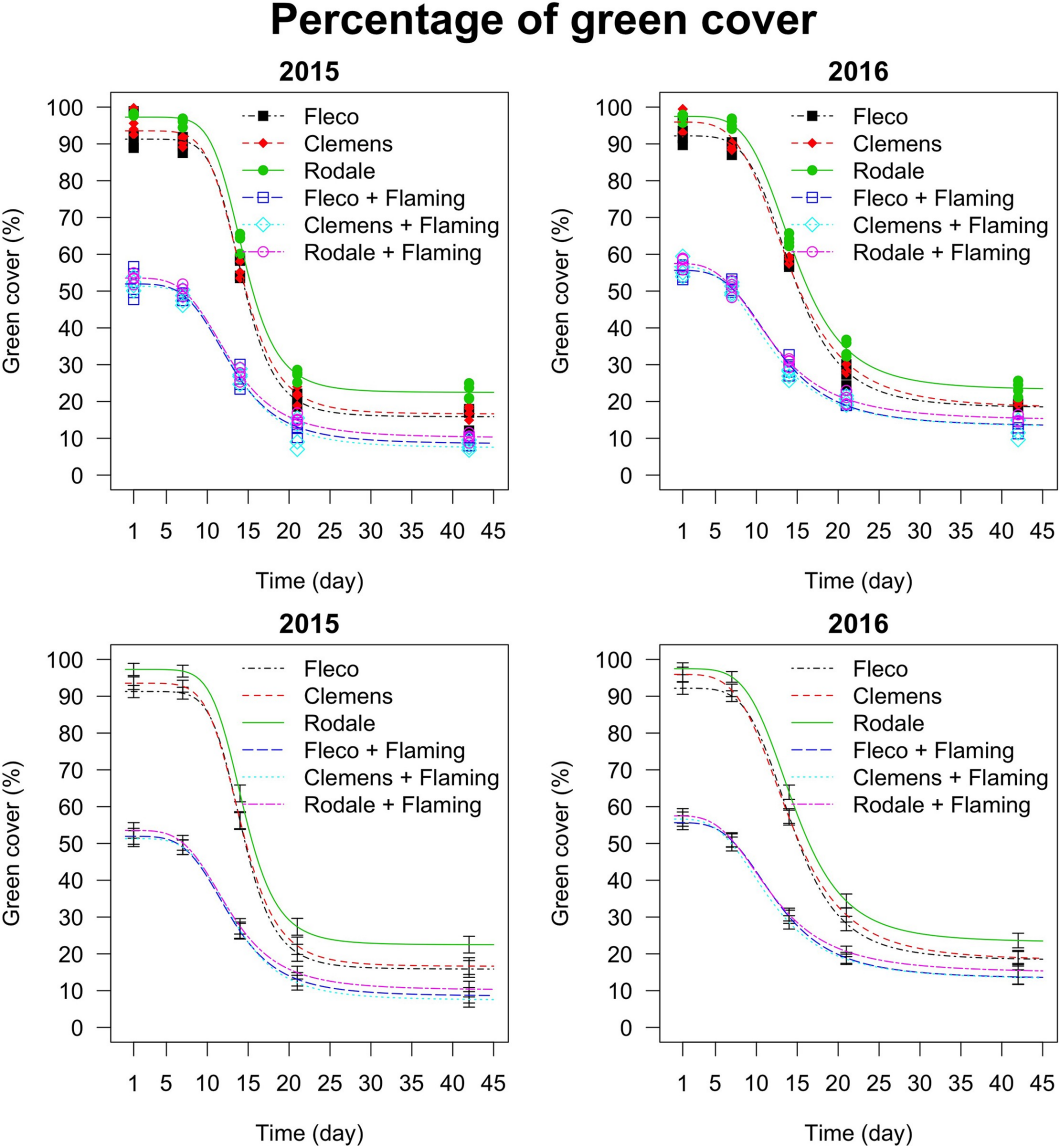
Percentage of green cover as affected by time (days after termination) and by the interaction between the type of roller (Fleco, Clemens and Rodale) and the flaming factor (if applied or not), in 2015 and 2016 growing seasons. All the data points and the model-based standard errors are reported, respectively, in the top and bottom graphs. The parameters of the curves (Eq 1) are reported in Table 2. Degrees of freedom: 96. The percentage of green cover before the termination was 100% in all plots.

The parameters of the four-parameter log-logistic model are reported in Table 2, and curves are plotted in Fig 7. The effect of flaming was statistically significant for all the parameters of the non-linear regressions. The upper limit of the curves (parameter *d*, Table 2) showed that when flaming was used in combination with rollers, the percentage of green cover one day after the termination was 1.8-fold and 1.7-fold statistically lower (95% CIs: 36.66, 42.04; 39.49, 44.96; 41.09, 46.40 for the Fleco, Clemens and Rodale roller, respectively, in 2015, and 34.06, 39.02; 36.58, 42.05; 37.45, 42.52, for the Fleco, Clemens and Rodale roller, respectively, in 2016) compared to that of the rollers used alone. At the lower limit of the curve (i.e. final percentage green cover), the difference in the percentage of green cover between the use of the rollers combined with flaming and the rollers used alone remained statistically significant (parameter *c*, Table 2), although this difference was lower than at the upper limit of the curves. This suggests that flaming improved the effectiveness of cover crop devitalisation compared to rollers used alone.

**Table 2:** Parameters and absolute effective times (i.e. response level refers to 100% of green cover observed before the cover crop termination) of the log-logistic function (Eq 1) modelling the green cover (%) as affected by time (days after termination) and by the interaction between the type of roller (Fleco, Clemens and Rodale) and the flaming factor (if applied or not), in 2015 and 2016 growing seasons. The nonlinear regression is plotted in Fig 7.

Treatment	Parameters				Absolute effective time	
	2015					
	*b* (±SE)	*c* (±SE)	*d* (±SE)	*e* (±SE)	ET_50_	ET_15_
Fleco	7.17 (0.94)	15.85 (1.17)	91.29 (0.84)	14.26 (0.15)	14.65 (0.17)	NA
Clemens	6.44 (0.78)	16.61 (1.19)	93.55 (0.87)	14.14 (0.15)	14.74 (0.18)	NA
Rodale	7.05 (0.84)	22.47 (1.17)	97.29 (0.84)	14.40 (0.16)	15.55 (0.25)	NA
Fleco + Flaming	4.33 (0.64)	8.49 (1.17)	51.94 (1.09)	12.83 (0.44)	6.32 (0.77)	19.157 (1.08)
Clemens + Flaming	4.53 (0.76)	7.40 (1.18)	51.32 (1.09)	13.20 (0.43)	6.13 (0.90)	18.633 (0.98)
Rodale + Flaming	4.16 (0.56)	10.13 (1.19)	53.54 (1.07)	12.70 (0.44)	7.09 (0.66)	20.876 (1.41)
	2016					
Fleco	4.88 (0.40)	18.27 (1.10)	92.182 (0.83)	14.35 (0.19)	15.21 (0.21)	NA
Clemens	4.09 (0.35)	18.13 (1.16)	95.937 (0.99)	14.00 (0.23)	15.31 (0.24)	NA
Rodale	4.74 (0.35)	23.14 (1.12)	97.474 (0.81)	14.59 (0.20)	16.45 (0.28)	NA
Fleco + Flaming	3.56 (0.38)	13.17 (1.16)	55.646 (0.96)	12.46 (0.44)	7.35 (0.50)	29.80 (2.96)
Clemens + Flaming	3.29 (0.33)	12.97 (1.19)	56.622 (0.98)	11.71 (0.44)	6.94 (0.43)	29.30 (2.98)
Rodale + Flaming	3.24 (0.33)	14.80 (1.16)	57.491 (1.01)	11.91 (0.47)	7.38 (0.47)	62.47 (10.58)

b is the slope of the function at the inflection point; c is the green cover (%) at the end of the experiment; d is the green cover (%) at 1 day after termination; e is the time (days) corresponding to 50% green cover between the upper and the lower limits of the curve (at the inflection point); SE is the standard error. Residual standard error: 2.29 and 2.02 for 2015 and 2016, respectively. Degrees of freedom: 96. ET_50_ is the absolute effective time (days) needed to achieve 50% of green cover. ET_15_ is the effective time (days) needed to achieve15% of green cover (i.e. 85% of devitalisation of the cover crop). NA: estimation not available

In both years, the absolute effective times (i.e. response level refers to 100% of green cover observed before the cover crop termination) showed that 50% of green cover, corresponding to 50% of devitalisation of the cover crop, was achieved about one week after termination in the flamed plots, irrespective of the type of rollers. On the other hand, when the roller crimpers were used alone, 50% of devitalisation was achieved only after about two weeks (ET_50_, Table 2), with a one day (significant) delay with the Rodale compared to the other two rollers.

In 2015, 15% of green cover, corresponding to 85% of the devitalisation of the cover crop, was reached about 19 days after termination (no statistical differences between type of rollers) when all the rollers combined with flaming were used. In 2016, when the Fleco and Clemens rollers were used in combination with flaming, 85% of devitalisation of the cover crop was achieved about 29 days after the termination, whereas when the Rodale roller was used in combination with flaming, 85% of devitalisation was achieved after about 62 days. When the roller crimpers were used alone, 15% of green cover was never achieved (this percentage was lower than the lower limit of the curves) in either of the two years (ET_15_, Table 2).

For the Fleco and Rodale rollers used alone, the time needed to reach 50% of green cover was statistically different between 2015 and 2016, with a slightly higher time in 2016 than 2015. For the same parameter, the Clemens roller used alone did not show any statistical differences between 2015 and 2016. For each type of roller combined with flaming there were no significant differences between 2015 and 2016 in terms of time for achieving 50% of green cover. However to devitalize 85% of cover crop plants, the time needed in 2016 was statistically higher for all the combinations between rollers and flaming compared with 2015.

In order to prevent the risk of yield reductions due to early-season depletion of soil water, stand losses and poor growth of crops following cover crops, Ashford and Reeves [10] recommended terminating cover crops three weeks before planting the cash crop as a standard practice. Delays in terminating the cover crop can decrease the time interval between rolling and planting the cash crop, and can also create problems of cover crop residue management during planting. Optimum residue conditions for planting a cash crop are usually reached three weeks after termination, when the residue becomes dry, crisp, brittle, and easy to penetrate with the cutting tools of the no-till planter/transplanter [1]. In our experiment, the predicted percentage of green cover (i.e. the percentage of cover crop not yet dead) three weeks after termination, was 13.09% (±0.92%), 12.16% (±1.01%) and 14.89% (±0.88%) for the Fleco, Clemens and Rodale rollers, respectively, used in combination with flaming, and 20.29% (±1.16%), 22.19% (±1.19%) and 27.35% (±1.15%) for the Fleco, Clemens rollers, respectively, used without flaming in 2015. In 2016, the predicted values were 18.91% (±0.75%), 18.53% (±0.71%) and 20.67% (±0.72%) for the Fleco, Clemens and Rodale rollers, respectively, used in combination with flaming, and 28.23% (±0.99%), 30.59% (±0.98%) and 34.35% (±0.96%) for the Fleco, Clemens rollers, respectively, used without flaming. Thus, in our conditions, flaming combined with roller crimpers speeded up the cover crop biomass desiccation, which after termination acts as mulch for weed management, and also creates the best conditions for cash crop establishment.

Mirsky et al. [3] suggested an economic threshold for cover crop control of 85% suppression, based on the industry standard for chemical herbicides. In our study 85% cover crop devitalisation was achieved only when the rollers were used in combination with flaming. In 2015, 85% of devitalisation was achieved about three weeks after cover termination. In 2016, this level of desiccation was achieved four weeks after termination. When the rollers were used alone, the maximum devitalisation of cover crops occurred 42 days after termination, ranging from 72% to 82%, with the lowest percentage of devitalisation estimated when the Rodale roller was used (Table 2). In 2015, the maximum percentage of devitalisation (about 93%) was achieved 43 days after termination when the rollers were used in combination with flaming.

None of the methods were able to reach the 100% of cover crop suppression, but in some cases even a 10–15% lower rate would not represent a real economic risk for the following cash crop. Especially when, as in our case, the growing season of the cash crop is not suitable for the growth of re-sprouted winter cover crops.

### Machinery performance and costs

Machinery performance and costs are reported in Table 3. The Fleco roller resulted in the highest static ground pressure, followed by the Clemens and the Rodale rollers. It should be taken into consideration that, during the operation of the rollers, the dynamic pressure applied on the ground is greater than the static pressure. Indeed, by maintaining constant the ground contact surface, an increase of the static load and forward speed will result in an increased dynamic load, leading to a higher dynamic ground pressure [35]. The highest working width of the Rodale roller determined, at the same forward speed, higher theoretical and effective field capacities, a lower theoretical field time, turning time, and time to refuel the tractor compared to the other rollers. Oppositely, the Fleco roller had the lowest working width; consequently, the trend of the above-mentioned operative parameters was opposite. The Clemens roller operative parameters were intermediate. Time to refuel the tractor was higher for the Fleco roller because it showed a higher theoretical field time and a higher power supplied by the tractor for towing a 1700 kg roller, which required refuelling the tractor more frequently. Machine adjustment time (including plugging and unplugging) was higher for the Clemens compared to Rodale and Fleco rollers. This was determined by the time spent for setting working width and connecting hydraulic plugs, which were not required for the Rodale and Fleco rollers. By summing all operative times, the Rodale resulted the roller with the lowest total time, whereas it showed the highest effective field capacity. The Fleco roller showed the highest field efficiency and the lowest effective field capacity. The flaming machine required a lower forward speed compared to rollers, thus determined higher total times and field efficiency, but a lower effective field capacity.

**Table 3:** Roller crimpers and flaming machines performance and costs estimation. All machines were used coupled with a New Holland TL100 tractor.

Performance				
	Fleco	Clemens	Rodale	Flaming machine
Ground pressure (MPa)	12.09	4.66	1.36	-
Forward speed (km h^-1^)	10.0	10.0	10.0	1.1
Working width (m)	1.82	1.98	2.88	2.00
Theoretical field capacity (ha h^-1^)	1.82	1.98	2.88	0.23
Theoretical field time (h)*	0.55	0.51	0.35	4.37
Turning time (h)*	0.17	0.12	0.08	0.12
Time to refuel the tractor and/or replace empty LPG tanks (h)*	0.005	0.004	0.003	0.127
Machine adjustment time (includes plugging and unplugging) (h)	0.17	0.25	0.17	0.25
Total time (h)*	0.89	0.88	0.60	4.86
Field efficiency*	0.62	0.57	0.58	0.90
Effective field capacity (ha h^-1^)*	1.12	1.14	1.68	0.21
Costs				
Cost per hour (€ h^-1^)*	38.31	35.27	34.60	70.29
Total cost per use (€ ha)*	34.22	30.94	20.60	341.62

*Time to conduct the operation in a hypothetical area of 10,000 m^2^ (30.00 m wide and 333.33 m long).

The cost per hour and the total cost per use of the Rodale roller was the lowest, whilst it was the highest for the Fleco roller. Generally, compared with rolling, flaming was noticeably the most expensive operation.

The different construction features (e.g. type and arrangement of blades) and ground pressure of the rollers (2.6 and 8.9 fold higher in the Fleco than in the Clemens and Rodale rollers, respectively) did not seem to affect the cover crop devitalisation. In fact, when the rollers were used alone without flaming, only a one-day delay was estimated (statistically significant) to achieve 50% devitalisation with the Rodale roller compared to the Fleco and Clemens (Table 2). At 42 days after the cover crop termination, the percentage of cover crop biomass that remained green was lower (6% in 2015 and 5% in 2016) when the Fleco and Clemens rollers were used, compared with the Rodale roller (parameter *c* in Table 2), likely without any noticeable effect on the sowing date of the cash crop. When the effect of flaming was added to the effect of rolling, the differences in the level of cover crop devitalisation became significant from an agricultural management point of view as well.

The high operational costs of flaming could be affordable in case of highly remunerative crops where alternative methods for termination of cover crops are not widely available, especially in herbicide-free systems, as for instance fresh market organic vegetable crops such as tomato, eggplant, sweep pepper, cabbage etc. that might be profitably grown in no-till conditions. In fact, the results of this study show that flaming used in combination with rollers actually increased the effectiveness of roller crimpers and sped up the cover crop termination in no-till soils. According to Kornecki et al. [1], this aspect could be of paramount importance, as any delay in terminating the cover crop can narrow the window between rolling and planting the cash crop and can also create problems during planting due to high biomass and humidity of cover crop residues. This could determine a late planting of the cash crop, which could compromise cash crop yield [1]. Note that a large amount of the variable costs for conducting flaming was due to the high cost of the LPG in Italy (2.27 € kg^-1^), and that flaming may be less expensive in countries where the LPG (or propane) would costs less (e.g. in the USA the propane cost is equivalent to 0.48 € kg^-1^).

### Cover crop height and biomass

The mean dry biomass and height of cover crop mixture at the time of termination are reported in Table 4. The height of the pea and barley in 2015 was not statistically different from that of 2016 (p-values = 0.07 and 0.20 for pea and barley, respectively). The dry biomass of the pea in 2015 was statistically higher than in 2016 (p-value = 0.00), whereas the dry biomass of the barley was similar in the two years (p-value = 0.17). The total dry biomass of the cover crop mixture (pea plus barley) was also similar between years (p-value = 0.97).

**Table 4:** Mean height and dry biomass, and standard errors of the cover crop mixture in 2015 and 2016 growing seasons.

Cover crop per year	Height (±SE) (m)	Dry mass (±SE) (Mg ha^-1^)
Pea—2015	1.01 (0.02)	1.94 (0.15)
Pea—2016	1.08 (0.02)	0.76 (0.15)
Barley—2015	1.01 (0.02)	6.61 (0.57)
Barley—2016	0.98 (0.02)	7.76 (0.57)
Pea plus Barley—2015	-	8.55 (0.50)
Pea plus Barley—2016	-	8.53 (0.50)

Least Squares Means degrees of freedom = 10.2, 11.8, 12, 12, and 12 for pea dry biomass, barley dry biomass, pea height, barley height, and the pea plus barley dry biomass, respectively.

The height and dry biomass of the cover crop mixture were similar in 2015 and 2016. However, the dry biomass of the pea alone was lower in 2016 than in 2015, suggesting that a lower number of plants grew during this growing season [36]. In 2016, pea growth was probably hampered by the competitive ability of barley, which requires a lower temperature than pea for vegetative growth and is characterized by a faster growth rate [36, 37]. In 2016, the average temperature in the initial months of the cover crop growing season (from October 2015 to January 2016) was lower than in 2015 (from October 2014 to January 2015) (Figs 1 and 2). Moreover, the rainfall from November 2014 to January 2015 was higher than from November 2015 to January 2016 (Figs 1 and 2). In 2016, both temperature and precipitation conditions probably led to a higher availability of N in the soil compared to 2015, thus promoting the rapid growth of barley [38, 17]. When the barley gains a competitive advantage in terms of growth during the initial stages of the growth cycle, this advantage remains for the rest of the growth cycle as well [39]. The higher proportion of pea in the mixture observed in 2015 (Table 4) could explain the slightly higher termination percentages observed in the first year as compared to 2016. Indeed, barley is well known to be harder to kill by roller crimper than pea, due to its high tillering capacity, which makes it very easy for barley plants to survive mechanical termination and re-sprout. Despite the differences in the pea dry biomass, the cover crop mixture showed a steady high aboveground biomass accumulation in both years, which confirms the well-known high complementarity between these species [40].

The cumulated rainfalls from April to June 2016 were much higher than in 2015. This was probably why, when the Clemens and Rodale rollers were used without flaming, 50% of cover crop devitalisation was achieved faster in 2015 than in 2016. The higher water availability occurred in 2016 probably enabled the cover crop plants to survive longer than in the year before. This delay in termination was more evident when rollers were used in combination with flaming, when 85% of devitalisation was achieved faster in the year when the rainfall was lower (2015), whereas the cumulated rainfall did not influence the time needed to achieve 50% devitalisation when rolling was combined with flaming.

The canopy of the cover crop mixture at the time of termination was quite tall (about 1 m) and presented a high dry biomass (8.5 Mg ha^-1^). Such high biomass is not easy to dry in a short time, especially when many rain events occur. Kornecki and Price [12] achieved a cover crop devitalisation percentage ranging from 94% to 97% three weeks after termination, however the dry biomass of the cover crop at the time of termination (5.5 Mg ha^-1^ on average) was lower than that of our experiment, and the cover species was different (rye, *Secale cereale* L.). Kornecki et al. [11] found at least 90% mortality of rye plants two weeks after rolling, with a dry biomass at the time of termination of 7.8 Mg ha^-1^ and a non-rolled rye control of 75% mortality.

## Conclusions

The use of roller crimpers in combination with flaming for terminating cover crops improved the effect of the roller crimpers used alone, and more importantly, shortened the time needed to achieve cover crop desiccation compared with rollers used alone. This is very important in order not to excessively delay the time needed for sowing/transplanting the cash crop. Flaming combined with roller crimpers could therefore be used as an alternative to chemical herbicides in all agricultural systems where the latter are forbidden or intended to be reduced.

Our study confirms that the high costs for implementing flaming in addition to rolling for terminating cover crops may hamper a wider adoption of no-till cover-crop based strategies. Farmer’s aim is earning, and the use of chemical herbicides is less expensive also because the application is faster. However, in Europe the future banning of some types of chemicals, deemed dangerous to health (e.g. glyphosate), should focus the attention on non-chemical alternatives. Probably, an inducement to adopt flaming could be the payment of incentives to farmers, which avoiding chemicals, could contribute to the improvement of environmental conditions.

Further research is needed to identify different roller crimpers designs that can adapt to specific environmental conditions and cover crop characteristics. However, in order to reduce the costs for farmers when buying different types of equipment for each combination of cover crop per pedo-climate, flaming could still be a viable way to increase the effectiveness of roller crimpers.

Further studies would be useful to investigate the effect of flaming in combination with other types of roller crimpers on other cover crop species, at different growth stages and in different pedo-climatic conditions. A new prototype of a combined machine, which could simultaneously conduct rolling and flaming, would likely reduce at least the costs of the operation and boost the large-scale adoption of this crop management system.
